# Camp Aesthetics as an Ethics of Transnormative Care: Challenging the Seriousness of Nursing From the Perspective of Latin American Travesti‐Trans Theory

**DOI:** 10.1111/nup.70128

**Published:** 2026-10-06

**Authors:** Yeymi Acevedo Ninco, Marcos Venicios de Oliveira Lopes, Viviane Martins da Silva, Reinaldo Gutiérrez Barreiro, Dave Bergeron, Marli T. Gimeniz Galvao

**Affiliations:** ^1^ Graduate Program in Nursing Federal University of Ceará (UFC) Fortaleza Brazil; ^2^ Department of Nursing Universidad Surcolombiana Neiva Colombia; ^3^ Department of Health Sciences Université du Québec à Rimouski (UQAR) Rimouski Canada

**Keywords:** 'camp'aesthetics, conceptual analysis, nursing philosophy, transnormative care, Travesti identity, Travesti theory

## Abstract

This article argues that the ‘politics of seriousness’ in nursing operates as a cis‐normative control mechanism that imposes sterile professional distances. To address the care of transvestite‐trans people, it proposes—drawing on the epistemologies of the Global South and through Latin American Transvestite‐Trans Theory (LATTT)—the adoption of an aesthetic of care based on Camp: a form of care that exalts the ‘*Trava*’, celebrates Affect and the Art of transvestite‐trans self‐determination, and replaces the hierarchical clinical protocol with ‘*Nostredad*’. To this end, a critical analysis was conducted using a theoretical framework that integrates Walker and Avant's model with Wayar's Latin American Transvestite‐Trans Theory. This contributed to the development of new theoretical concepts applicable to the practice of nursing care for transgender people. We conclude that transnormative care is truly ethical and must therefore be ‘unserious’: it must be exaggeratedly affective, complicit, and deeply relational to dismantle systemic violence.

## 1 Introduction

Trans people and Travesti‐trans people face healthcare systems organised around cisnormative, biomedical, and authoritarian assumptions about the body, gender, legitimacy, and healthcare (Gonçalves et al. 2026; Hughto et al. 2015; Reisner et al. 2016; White Hughto and Reisner 2016; Winter et al. 2016). In various settings, studies have documented barriers such as a lack of knowledge among healthcare staff, incorrect use of gender terms, disrespect for chosen names and pronouns, restricted access to diagnosis, institutional transphobia, fragmented services, and people forgoing care out of fear of discrimination (Cicero et al. 2019; Gonçalves et al. 2026; Kcomt et al. 2020).

Although guidelines that affirm transgender identity and patient‐centred models have contributed significantly to improving clinical practice through respectful communication, gender‐affirming interventions, and depathologizing approaches (Coleman et al. 2022; Robles et al. 2022), their scope and application are limited by multiple factors. These include political and ideological structures and inadequate healthcare systems, as well as deep‐seated socio‐emotional and epistemic barriers that shape how the lives of transgender and Travesti‐transgender people are perceived within healthcare institutions (Gonçalves et al. 2026; Reisner et al. 2016).

Respectful language, the correct use of pronouns, and access to gender‐affirming care are essential. However, these may prove insufficient when care continues to be structured around distance, neutrality, pathologization, and institutional respectability (Berdida 2026; Carel and Kidd 2014; Gonçalves et al. 2026).

This article analyzes these norms through the concept of 'serious nursing', clarifying that we are not referring to nursing as a whole, nor to the intentions of individual nurses. The traditional or 'serious' nursing we criticise is neither an unquestionable monolith nor a caricature of the profession or of individual professionals. Rather, we are referring to an institutionalised form of professional seriousness shaped by biomedical authority (Menéndez 2003), historical structures of patriarchal and positivist power (Reverby 1987; Hopkins Walsh et al. 2022).

In these settings, professional conduct serves as a framework of seriousness through which the nursing profession regulates credibility, physical presence, and a sense of belonging (Berdida 2026), projecting an image of emotional neutrality and clinical restraint. Their practice is often constrained by rigid protocols and a medicalized division of labour that prioritises emotional impartiality over relational vulnerability (Castro and Erviti 2015).

As feminist scholars and critics of nursing have long argued, the demand for 'emotional neutrality' and rigid clinical objectivity was originally a patriarchal mechanism designed to subordinate the caring professions to the biomedical model (Reverby 1987; Kagan et al. 2014). The practice of nursing has never been divorced from the political, economic, gender, and institutional forces that shape care relationships within the framework of the care economy (Dillard‐Wright 2022; Dillard‐Wright and Shields‐Haas 2021; Hopkins Walsh et al. 2022).

Therefore, this critique of 'seriousness' draws on this rich tradition of feminist, anti‐colonial, and queer research in nursing, which has historically resisted the sterilisation and alienation of care (Graziano 2026; Melino et al. 2024; Runtung 2026; J. B. Smith et al. 2023; Stake‐Doucet 2023; Hopkins Walsh et al. 2022).

We recognise the fundamental advances achieved by contemporary clinical guidelines, which affirm transgender people and promote patient‐centred models that seek to ensure respectful treatment and regulate interactions within the healthcare system. However, their implementation is shaped by hierarchical power dynamics that standardise procedures and shape conditions and attitudes dictated by bureaucracy and preexisting neoliberal policies (Spade 2011).

It is here that we suggest 'Camp' as an alternative, so that it transcends the bureaucratic checklist of affirmative guidelines and offers an ethical and aesthetic framework that actively embraces shared vulnerability—in Wayar's words, seeking '*Nostredad'* (Wayar 2018), which protocols alone cannot achieve.

Rules regarding professional boundaries, distance, and professionalism are often presented as guarantees of rigour and safe therapeutic interaction (Hungerford 2025). However, they can also perpetuate exclusion when they demand that nursing remain detached from the aesthetic, emotional, and political ways in which Travesti and transgender people create lives worth living (Dillard‐Wright 2024, 2025; Pierce 2020).

This critique is situated within the institutional contexts of health care in Latin America, particularly those shaped by colonialism and biomedicine (Gonçalves et al. 2026; Menéndez 2003, 2020). In contexts where the lives of Travesti and transgender people may be viewed solely as clinical identities, there is an urgent need to recognise them as political, aesthetic, and epistemological forms of survival.

The term 'Travesti', as used in this article, is not interchangeable with the generic English‐language term 'trans'. It refers to a Latin American political subjectivity that is specific to the region and has been historically asserted, forged through resistance to medical, police, colonial, cisheteronormative, and patriarchal violence (Martínez and Vidal‐Ortiz 2021; Pierce 2020; Wayar 2018).

To rethink nursing care from this perspective, we turn to ‘Camp’. Following Sontag, 'Camp' is often understood as an aesthetic sensibility characterised by exaggeration, theatricality, artifice, and a love of the unnatural (Sontag 1964). However, this article does not introduce 'Camp' in nursing as a decorative or stylistic concept. Instead, it asks what happens when 'Camp' is interpreted through the Latin American Travesti‐Trans theory (LATTT), in which aesthetics, affect, and self‐reinvention function as technologies of survival rather than as superficial expressions (Cleto 1999; Darouiche 2025; Debia 2020; Pierce 2020).

Latin American travesti‐trans theory, particularly the work of Marlene Wayar, provides the conceptual foundation for this reinterpretation. Concepts such as '*Origen Trava, Affect, Art, Rage, and Nostredad*' allow us to understand travesti‐trans self‐construction not as a pathology, deviation, or clinical problem, but as an aesthetic and political practice of creating life (Daix 2021; Darouiche 2019, 2025; Wayar 2018).

The aim of this article is, therefore, to develop Camp aesthetics as a transnormative ethics of care in nursing. Through a critical adaptation of Walker and Avant's concept‐derivation approach (Walker L Olszewski 2018), a dialogue is established between Camp aesthetics and Latin American Travesti‐trans theory.

The aim is to identify the main concepts, background, defining attributes, and implications of an alternative form of nursing care that is less 'serious', more relational, and epistemically responsible. We argue that this type of care does not sacrifice clinical rigour; rather, it reconfigures that rigour beyond affective neutrality, orienting it toward relational responsibility and epistemic justice (Berdida 2026) and '*Nostredad'*, as a collective, relational form of resistance (Wayar 2018).

Before continuing, it is important to establish our starting point. Our team writes from the perspective of public universities throughout Latin America and Quebec (Canada); two of us work at universities located outside major metropolitan areas, a situation that shapes our focus on territorial, institutional, and epistemic margins in the fields of health and nursing.

Our perspectives are based on research and teaching in nursing, global health, and the health of minority groups who find themselves in vulnerable situations due to colonial, heteronormative, and institutional structures. We approach this analysis not as neutral observers, but as researchers concerned about violence epistemic historically generated by the biomedical disciplines (Santos 2014), including nursing. We seek to engage with travesti‐trans epistemologies through humility, relational responsibility, and attentive listening, thereby contributing to a form of nursing philosophy capable of learning from the political, aesthetic, and collective survival strategies of those who are relegated to the margins of clinical seriousness (Carel and Kidd 2014; Fricker 2007).

## 2 Theoretical Framework: From 'Camp Sensibility' to Travesti‐Trans Epistemologies

This section expands on the conceptual foundations established in the introduction, clarifying the theoretical framework on which the article is based: from 'Camp' as an aesthetic sensibility, through Travesti‐trans epistemologies as political theories of survival, to nursing care as a potential arena for ethical reconfiguration.

### 2.1 Camp Beyond Aesthetic Sensibility

Sontag's description of 'Camp' serves as the conceptual starting point for this critical analysis, as it defines a sensibility linked to artifice, stylisation, exaggeration, theatricality, and a particular relationship with seriousness (Sontag 1964). In this formulation, 'Camp' does not merely reject seriousness; rather, it destabilises the hierarchy that makes seriousness appear more truthful, legitimate, or valuable than artifice, representation, and exaggeration.

Cleto's work allows us to interpret 'Camp' from the perspective of queer aesthetics and performance, where authenticity lies not in naturalness, but in the ability to expose, disrupt, and rework normative codes (Cleto 1999). This distinction is important for the philosophy of nursing, as this article uses it as a philosophical lens to examine how legitimacy is attributed to bodies, emotions, appearances, and modes of self‐presentation.

From this perspective, Camp calls into question a fundamental assumption of clinical seriousness: that what is natural, stable, biologically legible, and professionally neutral is more reliable than what is aesthetic, affective, exaggerated, or self‐created. This theoretical shift sets the stage for the article's central thesis: what clinical institutions may dismiss as artificial, excessive, theatrical, or frivolous is fundamental to the way in which Travesti and transgender people create livable worlds.

### 2.2 Travesti‐Trans Epistemologies as a Plural Field

Although the Latin American Travesti‐Trans Theory (LATTT) (Wayar 2018) provides the central conceptual foundation of this article, the theoretical framework from which it is written also draws on other authors. Rizk positions Latin American theorising on travesti‐trans as a field of political thought linked to feminist and collective struggles (Rizki 2025). This supports the decision to approach *travesti*‐trans thought as a political and epistemological tradition, rather than as a marginal identity‐based discourse.

Martínez et al. describe 'locking in knowledge' as a form of knowledge construction centred on the *Travesti* that breaks with dominant educational and epistemic systems (Martínez and Vidal‐Ortiz 2021). This idea is central to this manuscript, as it allows us to understand Travesti‐trans knowledge as a source of theory.

Cabral's critique of biomedical normalisation helps clarify how clinical institutions can transform bodily and gender diversity into objects of classification, correction, or administrative management (Cabral et al. 2009). Pierce shows how trans and *Travesti* cultural production in Latin America mobilises and embodies performance as a form of resistance (Pierce 2020), reinforcing the idea that aesthetics and embodiment are not secondary.

While Marlene Wayar's conceptualisation of the *Origen Trava*, Art/Artifice, Affect/Rage, and *Nostredad* forms the cornerstone of this theoretical derivation, this framework emerges from a pluralistic and polyphonic Latin American intellectual tradition. This is also true of the pioneering work of the Argentine *Travesti* activist and theorist Lohana Berkins, who warned against 'epistemic travesticide' (Berkins 2007), referring to the systematic erasure of knowledge about *Travesti* by academic and biomedical institutions (Simonetto and Vidal‐Ortiz 2025).

Her activism demonstrated that being a *Travesti* is not merely a gender identity, but a profound political stance—a broad epistemology of survival that demands a redistribution of power and knowledge in health care. Similarly, Mauro Cabral Grinspan's work highlights how biomedical normalisation inherently seeks to correct and pathologize bodily diversity (Cabral 2006; Maffia et al. 2003), which underscores the need for models of care that resist normative assimilation.

Within this pluralistic context of the Global South, we must reexamine the traditional conceptualisation of 'Camp'. Although Sontag defined it as 'a love of the unnatural and the exaggerated', (Sontag 1964), While an apolitical aesthetic sensibility may exist, LATTT radically politicises it. In contexts of extreme institutional violence and marginalisation, artificiality and aesthetic exaggeration are technologies of survival: popular technologies of resistance.

Therefore, when applied to the clinical setting, 'Camp' aesthetics ceases to be purely visual and becomes deeply ethical. It requires nurses to recognise, validate, and participate in this aesthetic resistance, transforming the clinical space from a place of medical scrutiny into a shared trench: *Nostredad*.

'*Nostredad'*, understood as a form of 'us', offers an alternative to clinical 'otherness' by reimagining care as a relationship co‐constructed through recognition, vulnerability, and collective survival (Rizki 2025; Wayar 2018).

Taken together, these authors allow us to frame *Travesti*‐trans epistemologies as plural, embodied, activist, aesthetic, and—above all—political. Far from serving merely as a contextual backdrop, this field provides the theoretical framework necessary to interpret Camp as an ethical resource for nursing care.

### 2.3 The Conceptual Coordinates of Wayar: *Origen Trava*, Affection, Art, Rage, and *Nostredad*

The concepts of *Origen Trava*, Affection, Art, Rage, and *Nostredad*, proposed by Wayar (2018, 2021), provide the analytical framework for the conceptual derivation developed in the following section. These concepts are abstract categories linked to lived experience; they arise through the body, memory, violence, 'collective survival, and the refusal to be reduced to a pathology.

'*Origen Trava*' challenges biomedical narratives that seek the 'cause' or origin of gender variation in pathology, diagnosis, or developmental anomalies (Wayar 2018). Meanwhile, it situates the *Travesti*‐trans experience within the context of the histories of coloniality, gender regulation, violence, desire, and self‐determination. For nursing, this concept raises a philosophical question: Why do clinical systems often attempt to explain, authorise, or authenticate trans existence before addressing it?

Wayar asserts a pre‐Columbian 'origin' of travestidad: the '*Origen Trava'*. In this framework, the lives of *Travestis* and trans people are torn between the 'internal truth of gender'—disconnected from 'reality'—and the 'external reality of the sexualised body'. In this way, it not only connects cross‐dressing to a long‐standing, indigenous, and violent past that has shaped trans lives and histories, but also links it to the axis of racialization, thereby exposing the coloniality of gender (Wayar 2018).

At the same time, 'affect' defines the emotional and political textures of survival for *Travesti* and trans people. Wayar addresses rage, fear, rage, tenderness, grief, splendour, and collective care—not as private emotions, but as a response to worlds that have made the lives of *Travesti* and trans people precarious. The response is to create their work, their lives, and their bodies, mobilising emotions and affects that fuel a collective sense of protest against indifference and multiple forms of sex‐gendered and institutional violence—police, educational, and religious—that target the bodies and childhoods of *Travesti* and transgender people (Wayar 2018).

The indignation, fear, and rage Wayar speaks of translate into a 'Rage' that allows meanings to emerge and creates practices that enhance the artistic power of cross‐dressing—evident in its ability to forge and recreate itself through the body, emotion, thought, and desire. 'Rage' is an affective response to structural violence and a force capable of sustaining resistance (Wayar 2018).

In this regard, 'Rage' allows us to rethink affect not as something that must be neutralised in clinical encounters, but as a potential space for ethical and political understanding. In the field of nursing, this concept challenges the assumption that emotional neutrality is always the most ethical professional stance.

'Art' refers to the self‐creation of the body, identity, gesture, voice, style, and presence as a political and existential endeavour. Wayar (2018) frames the self‐construction of *Travesti* and transgender people as an embodied aesthetic practice, rather than as a failed attempt to approximate biological normality.

*Travesti*‐trans art consists of challenging the discourses and the perceptual and conceptual logics of binary thinking, thereby enabling alternative configurations of the body and other ways of living, coexisting, and being (Wayar 2018). This concept is fundamental to the article's analysis of Camp, as it reframes artifice and aesthetic exaggeration as practices of truth, survival, and freedom.

*'Nostredad'* provides the relational framework for this article. Wayar uses it to move beyond 'otherness' and toward a collective sense of 'us'. '*Nostredad'*, in its deepest sense, evokes the sense of being 'us'—a collective subjectivity forged in the shared, collective experience of marginalisation, resistance, and struggle, which builds art and cultures, with artisans who integrate theatricality, exaggeration, and extravagance into their lives—not merely to be noticed and felt, but as a way of life, as discourse made flesh.

Rizki helps situate this relational approach within a broader *Travesti*‐trans politics of coalition, survival, and feminist struggle (Rizki 2025). In the field of nursing, *Nostredad* does not imply a dissolution of ethical responsibility or professional accountability. Rather, it explores how care might be co‐constructed through recognition, reciprocity, and collective survival.

### 2.4 Toward a Transnormative Ethics of Care

Taken together, 'Camp' aesthetics and *Travesti*‐trans epistemologies allow us to rethink the ethical foundations of nursing care. Wright argues that nursing requires a radical imagination capable of resisting inherited disciplinary norms and envisioning care in a different way (Dillard‐Wright 2022). This supports our assertion that the question is not whether nurses should abandon rigour, boundaries, or safety, but whether rigour itself can be reconfigured so that it does not depend on emotional distance, cisnormative classification, or biomedical respectability (Dillard‐Wright 2024).

Berdida is particularly useful in this context, as she argues that professional conduct in nursing functions as a discipline of seriousness through which nursing regulates credibility, embodiment, and belonging (Berdida 2026). This allows us to link the article's critique of 'serious' nursing to a broader philosophical debate within the nursing profession regarding professional performance, moderation, respectability, and legitimacy.

The following section develops this argument from a methodological perspective through a critical adaptation of Walker and Avant's conceptual derivation approach. *Origen Trava*, Affect, Art, Rage, and *Nostredad* are used as conceptual coordinates to identify the antecedents, defining attributes, and consequences of transnormative care inspired by 'Camp'. In this way, the theoretical framework is not limited to a general philosophical reflection but becomes the basis for a structured conceptual derivation within the field of the philosophy of nursing.

## 3 Conceptual Derivation: Moving Clinical Seriousness Toward an Ethics of Care (Camp)

To frame Camp aesthetics as a framework for transnormative care, the 'concept derivation' methodology proposed by Walker and Avant was employed (Walker L Olszewski 2018). Regulatory mechanisms governing clinical 'seriousness'—which can end up excluding marginalised subjectivities—the referral was not carried out conventionally; rather, an epistemic hack was performed (Mignolo 2009). In keeping with the critical stance outlined above, we have adapted this framework through the lens of Latin American Travesti‐trans epistemology.

Let us recall that in the derivation of concepts, a concept is borrowed from another discipline when the literature is scarce or when there is a desire to adapt those concepts to the new field. In this critical adaptation, the evidence or concepts to be derived are not biomedical cases, but rather the survival strategies and the political and emotional experiences of the trans population in the Global South. Four analytical steps were followed for the derivation, which constituted the conceptual framework (see Table 1).

**Table 1:** Conceptual derivation matrix: deconstructing 'seriousness' toward transnormative care in nursing based on the LATTT.

Camp Base Concept (Wayar)	Serious Background (Precedes the concept—must be dismantled)	Critical Attribute at Camp (The New Lens)	Implications for the New Camp (Transnormative Practice)
1. *Origen Trava*	Biological Truth: Nursing focuses on the linear medical history, genetics, and the diagnosis of 'dysphoria'. The modified body is interpreted as 'falsehood' or pathology.	Epistemic disobedience, recognising the 'invention of the self' as a legitimate process rooted in desire, difference, and truth.	Ontological Validation: Nursing ceases to be the 'police of biological truth' and recognises self‐construction. The chosen name and identity are the first relevant part of the medical history.
2. Affection	Emotional Neutrality: Professionalism requires detachment. Anger, drama, or *Travesti* crying are interpreted as psychological instability or 'misconduct'.	Political Rage: Emotion is intense, dramatic, and vital. Anger is understood as a lucid and healthy response to systemic oppression, not as a symptom.	Emotional support as resistance: Care does not seek to 'calm' or 'normalise' emotion, but rather to channel that energy as a driving force for survival. Nursing that resonates with *Travesti* political rage.
3. Art	Body Modification: The focus is on 'curing' or helping someone 'pass' (appear cisgender). Cosmetic accessories are viewed as decorative frivolities.	The Body as a Canvas (Co‐Curated): The body is a work in progress. Aesthetics (makeup, wigs, fashion) are a 'Technology of the Self' and a lifeline.	Aesthetic Life Support: Nursing as a co‐creator of the work and the transition process. The patient's aesthetic elements are protected and maintained with the same rigour as a medical device.
4. *Nostredad*	Professional Boundaries: The relationship is hierarchical (Expert‐Patient). Individual autonomy and aseptic isolation ('Therapeutic Distance') are prioritised.	Emotional complicity—Breaking down the aseptic barrier. A collective subjectivity ('We') that brings people together and unites them. The tribe provides protection where the state fails.	Shared Trenches: Care Becomes a Political Alliance. Nursing breaks down the hierarchy to become part of the *Travesti*‐trans tribe/network in resistance to institutional hostility.

*Note:* Original work based on adaptations by Walker (2018) and Wayar (2018).

The first step was to select the main concept and the epistemic source. The 'literature' analysed here is the theoretical corpus by Wayar (2018). The source concepts were the 'Camp' aesthetic, re‐theorised through Latin American Travesti‐Trans Theory (LATTT). The theoretical and autobiographical corpus of trans activists Wayar and Berkins provided the foundation for an ethical approach that affirms trans identity—an approach currently lacking in English‐language biomedical literature (Berkins 2007; Wayar 2018).

In this case, the concepts were: the *Origen Trava*, Art, Affection, and *Nostredad* (see Table 1, Column 1), as counter‐hegemonic approaches to traditional nursing literature on trans care, which is dominated by pathologization and biologistic models that shape the discipline through whiteness, patriarchy, and capitalism (Hopkins Walsh et al. 2022).

As a second step, we identified the underlying factors of 'seriousness', understood as the preconditions that trigger the need for the Camp concept to emerge. These underlying factors are elements of cis‐heteronormative violence and epistemic injustice developed by 'serious' healthcare systems that give rise to clinical distance.

Through our analysis of the LATTT corpus, we identified the following factors preceding the *Origen Trava*: biomedical rigour, the search for biological truth, the imposition and classification of a 'normal‐abnormal' origin, pathologizing diagnoses, and sequential protocols and narratives that seek to control authenticity in the transition through gender and *Travesti*‐trans bodies (see Table 1, Column and Row 2).

About the background of the seriousness that gives rise to the need for 'Affect' as a Camp concept (see Table 1, [Column 2, Row 3]), the following were identified: emotional neutrality, the rationalisation of affect, obedience to rigid role structures, hegemonic power structures in neoliberal systems, and contextual disconnection and indifference toward trans realities. The system absorbs nursing professionals, who are accustomed to grey, cold spaces and bureaucratic processes that wear them down and cause them to lose their capacity for wonder and connection.

Similarly, after critically analysing the concept of Art, the following were considered as background factors: the idea of bodily correction, and the treatment of the cis‐heteronormative biological body as the authentic and true body (see Table 1, Column 2, Row 4). In 'serious' Latin American healthcare systems, clinical and social distancing is perpetuated by referring to and treating trans bodies as dysphoric bodies undergoing correction processes.

Regarding the background leading up to the concept of '*Nostredad*' (see Table 1, Column 2, Row 5), the following were considered elements of cis‐heteronormative violence: professional boundaries and distancing, ideologies that have shaped the discipline—such as whiteness, patriarchy, and capitalism—the dominance of individual care, and hierarchical power structures.

This second step made it possible to recognise how each of the serious issues that exist and arise in the day‐to‐day reality of trans interactions leads to a profound alienation from health care services and clinical settings. It also highlighted how the emotional detachment characteristic of traditional nursing contributes to the creation of a systemic failure that turns a 'Camp' intervention into a matter of survival.

The third step in the derivation process consisted of identifying the defining attributes—that is, the characteristics that most frequently manifest when the concept is present. In our case, what essentially defines Transnormative Care is artifice, emotional complicity, and aesthetics as a lifeline.

By intersecting nursing ethics with *Travesti* survival, we derive the critical attributes of each central concept. Thus, for *Origen Trava*—as a radical self‐determination of the trans gender that celebrates the collective survival of trans people from the margins and pre‐Columbian times (Wayar 2018)—epistemic disobedience was identified as the primary attribute (see Table 1, Column 3, Row 2): 'the invention of the self and the artificial', as an expression of affirmation (trans body, identity name, trava self‐determination, *Travesti*) as the authentic truth of the subject, not as a biological error to be corrected, but as a valid and legitimate site of knowledge, both historical and political.

For the concept of 'Affection', in contrast to the affective neutrality required by nursing and determined by intentional vulnerability, political love, and complicity, the attribute 'political rage' emerges (Ahmed 2014; Munt 2017; Wayar 2018) (See Table 1, Column 3, Row 3). Here, political anger against injustice is channelled, seeking to use emotions to foster self‐determination and self‐creation, and accepting Rage as a valid and protective emotion—a healthy response—rather than viewing it as a behavioural disturbance or a symptom.

Concerning the concept of Art, we recognise that, in the Global South, 'Camp'—through travesti‐trans theorisation—transcends superficial aesthetics to become a vital technology. Therefore, among the attributes of Art (artifice) (see Table 1, [Column 3, Row 4]), the following are included: 'the body as a canvas', aesthetic exaggeration, and intentional theatricality as a performative rupture (Pelúcio 2014), 'Artifice and Exaggeration', relying on the intentional use of hormones, makeup, procedures, glamour, humour, and hyperfemininity as protective shields against systemic violence and clinical sterility in the clinical encounter. These elements function as a communicative tool that highlights travesti‐trans art and creation—which is not about trying to fit in or appear to be something, but rather about simply being.

Ultimately, the concept of '*Nostredad*' emerges as the definitive synthesis of LATTT, arising when the strict boundary between the healthcare professional (the 'serious' one) and the pathologized/marginalised patient is broken down. Following conceptual analysis, *Nostredad* reveals 'affective complicity' as one of its defining attributes (see Table 1, Column 3, Row 5). Affective defiance against a utilitarian system that uses seriousness as a tool of oppression toward *Travestis* and trans people as well as healthcare professionals stems from shared emotions, radical relational equity, and the dissolution of clinical otherness (Wayar 2018).

As the fourth and final step in deriving the 'Camp' concepts from LATTT for application to nursing care, the implications were defined. That is, the outcomes that would result if the 'Camp' concepts were adopted and adapted to nursing practice. In other words, they represent the clinical transformation of transnormative practice.

If the nursing profession successfully adopts this 'Camp' ethic, the results will extend beyond mere medical compliance. The implications discussed here advocate for dismantling paternalism, improving experiences of affirming care and adherence to treatment, and creating safe spaces for trans and *Travesti* individuals—all of which are tangible clinical outcomes derived from philosophical concepts.

When nursing staff recognise the *Origen Trava* accordingly, they will validate the patient as the ultimate epistemic authority over their own body (Radi 2019) (see Table 1, Column 4, Row 2) Care will shift from a pathologizing, diagnostic approach to a dialogic and collaborative one, in which the lived experience of *Travesti* and transgender individuals and communities is considered sufficient and valid clinical evidence.

The clinical consequence of adapting Affect to care will be the transformation of the nurse into an emotional support figure who is approachable and capable of channelling political Rage, moving away from the role of a distant and decontextualised healthcare professional (see Table 1, Column 4, Row 3). This affective commitment would facilitate the emotional transition brought about by the historical trauma of institutional violence, fostering clinical trust and improving care through mutual respect rather than coercion.

Meanwhile, the introduction of art into the realm of care will result in the humanisation of the clinical encounter beyond biological parameters, offering 'aesthetic life support' (see Table 1, Column 4, Row 4). When nursing takes a genuine interest in the aesthetic appearance of *Travesti* and drag individuals, recognising and valuing their desire, effort, and autonomy in self‐creation—rather than dismissing it as irrelevant to medical care—it will become a co‐creator and advocate for that creation. Art in trans care would advocate for aesthetic elements (hormones, makeup, voice therapy, wigs, etc.), understanding them as technologies of trans survival.

Ultimately, the consequences of applying *'Nostredad*' to trans care will be that the care setting will cease to be a disciplinary institution and become a 'shared trench' of mutual protection. In this context, nursing shifts from observing and judging the actions of the Travesti‐trans patient to embracing the 'we' that permeates care—a state of relational equity that leads to greater epistemic justice and deconstructs pathologizing clinical perspectives.

The proposal, therefore, is that '*Nostredad*' allow nursing staff to become emotionally and affectively involved. That they become part of the 'tribe' that protects trans people, that they break down the hierarchy, and that they shift the dialectic and narrative from 'care' to 'co‐creation', while defending the self‐determination of *Travesti* and drag people's lives.

Table 1 is presented below, summarising the analytical process conducted according to Walker and Avant's framework. It identifies the four main Camp concepts through the LATTT framework, their background, the critical attributes derived from each concept, and finally presents the practical consequences or outcomes resulting from the adoption of these concepts in trans care in nursing. This matrix illustrates how transnormative care is not an incremental improvement on traditional care, but rather an epistemological break from care, resulting from a critical analysis of the LATTT.

### 3.1 Reflexivity in the Analytical Process

Given the regulatory and political dimensions of trans healthcare, it is necessary to reflect on our own analytical process during this conceptual shift. In applying Walker and Avant's framework to LATTT, we are constantly confronted with the methodological tension of 'toning down' the intensity and emotionality of *Travesti* thought to adapt it to academic standards in nursing.

Throughout the adaptation process, we intentionally resisted the temptation to tone down Wayar's 'Rage' or translate it into more docile and acceptable clinical terms, such as 'patient dissatisfaction'. Our analytical decisions—such as retaining the LATTT terminology in Spanish and prioritising grassroots activist literature over traditional biomedical journals—were deliberate strategies to ensure that epistemic authority remained in the hands of the *Travesti* community.

This deliberate stance was crucial to honouring the voices of Latin American theorists and the conceptualisation of '*Travar el saber*' as a political identity, ensuring that our conceptual derivation did not become yet another form of academic extractivism (Martínez and Vidal‐Ortiz 2021; Smith 2021).

### 3.2 The Structural Conditions of the Nursing Professional's 'Seriousness': Neoliberalism and Trans Healthcare

Although this analysis advocates for a substantial shift in nursing ethics regarding the care of transgender people, it is essential not to place the responsibility for clinical 'seriousness' on individuals. The emotional detachment we criticise is rarely a malicious or isolated choice on the part of healthcare professionals. Rather, it is a rational and often forced adaptation within contemporary Latin American healthcare systems.

Within the framework of neoliberal biopolitics in healthcare settings, nursing work is heavily commodified. Efficiency, a culture of auditing, and the standard protocol of short consultations and excessive workloads are prioritised over the time devoted to relationships (Peter et al. 2004; Traynor 2013).

The pressure resulting from staff shortages, rushed consultations, and fragmented care structurally stifles the ability to form emotional bonds (Peter et al. 2004). In this context, 'professional seriousnes' serves as a psychological defence mechanism against systemic burnout and automation in response to the institutional mandate to treat patients quickly.

When applied to transgender populations, these structural limitations are actively harmful. As Pearce (2018) points out, institutional dynamics—including pathologizing diagnostic pathways, rigid protocols, hierarchy and bureaucracy in power structures and processes, as well as a lack of funding—compel healthcare professionals to assume the role of bureaucratic gatekeepers (Dillard‐Wright 2022, 2025; Pearce 2018).

As is evident, these institutional dynamics—which shape the reality of healthcare professionals and care systems dominated by power structures and market forces—transcend borders. They reveal a reality that affects and troubles not only our Latin American context but is also a source of concern in the Global North (Pearce 2018).

Amid these crises in health care systems, a crisis of identity and status within the nursing profession has arisen—and this is no accident. It is the historical result of entrusting the profession (a predominantly female group) with the ethical imperative to care, while denying it the structural and economic power necessary to control how that work is carried out (Reverby 1987).

In Latin American public health systems, professionals with no training in caring for transgender people are often left to fend for themselves, relying on deeply entrenched cisnormative protocols, without institutional support or adequate tools. Thus, the 'seriousness' that marginalises transgender bodies is imposed from the top down.

Therefore, embracing the 'Camp' aesthetic and cultivating *'Nostredad*' cannot be merely an individual desire or ethical practice. They represent a collective and structural form of resistance—an epistemic strike against the neoliberal subordination of care. With this critical epistemic proposal, nursing has a field of action by choosing to inhabit the 'Camp trench', where it ceases to see a patient and instead sees a *Travesti* ally. There, it could seek ways to actively rebel against the colonial power structures in healthcare.

Speaking out against institutional violence, moving away from emotional neutrality and professional paradigms that create distance and fragment trans care—these are the conditions that pave the way for transnormative care.

## 4 Discussion: From Clinical Detachment to the Trenches of the 'Camp'

The derivation of 'Camp' aesthetics from Latin American *Travesti*‐Trans Theory (LATTT) poses fundamental challenges to the epistemological stance of contemporary nursing. Through a systematic analysis of the origins, attributes, and implications of *Origen Trava*, Art, Affect, and *Nostredad*, this study demonstrates that 'Camp' is not a superficial stylistic choice. It is an alternative approach to care, grounded in a solid ethical framework for transnormative care.

The traditional nursing paradigm for caring for vulnerable people is based largely on professionalism and existing clinical superiority, as well as on cultural competencies, trans‐affirmative checklists, and protocols that seek to address health inequalities. However, as noted in the theoretical framework, these approaches end up reinforcing the otherness they aim to bridge (Pearce 2018). They position the cisgender nurse as the normative standard and *Travesti* women as the 'other'—the one who is not recognised—who must be tolerated, studied, or accommodated within existing biomedical protocols.

In this context, the 'Camp' ethic discussed here proposes a substantial departure from this clinical superiority. Through *'Nostredad'*, nursing does not merely accommodate the trans patient. Rather, it actively engages with their shared vulnerability, breaking down the division between subject and object that defines formal institutional care and adapting the setting of the encounter and care to the 'shared trench'.

Furthermore, applying Wayar's concepts of Affection/Rage and Art/Artifice to the practice of nursing responds to the urgent call to decolonise healthcare ethics and the philosophy of nursing. These have long been based on Eurocentric and North American care frameworks, historically prioritising emotional regulation and objective rationality (Kagan et al. 2014).

These reflections focus on ethical care in deeply hostile environments that require diverse ways of politicising love and emotional solidarity in caregiving. For this reason, the survival and resistance strategies of Latin American *Travesti* women are celebrated. The aesthetic exaggeration of 'Camp' becomes a clinical tool that neutralises and mitigates institutional trauma, creating a safe environment in which one is allowed to be simply. Here, care and healing are mediated by artifice, extravagant emotionality, joy, Rage, and mutual recognition, rather than by absolute and sterile control.

In short, adopting a 'Camp' ethic of care is an act of epistemic rebellion. It challenges the neoliberal and biomedical constraints imposed on nursing professionals. It proposes that, to care for trans and *Travesti* populations effectively, nursing must dare to be frivolous and protect '*Nostredad'*.

## 5 Conclusion

'Seriousness in nursing' has long functioned as a regulatory barrier. In the care of trans and *Travesti* populations, it distances them from their lived experiences and their aesthetic survival strategies. By critically reinterpreting Walker and Avant's conceptual framework from the perspective of Latin American *Travesti*‐trans theory, this article presents 'Camp' as a vital ethical framework for transnormative care.

Beyond the limitations of standard care guidelines, the Camp encourages nursing staff to move beyond their emotional detachment, professional neutrality, and patriarchal and colonial attitudes toward the affective front lines of '*Nostredad'*. This does not mean sacrificing clinical rigour, but rather recognising that, in contexts of systemic hostility, politicised artifice and affective complicity are necessary protective measures.

By embracing the *Origen Trava*, nursing can begin to repair the historical damage caused by epistemic violence, transforming the clinical encounter from a site of biomedical regulation into a shared space of political resistance, epistemic justice, and healing.

## Author Contributions

**Y.A.N.** conceptualised the study, performed the theoretical derivation, and drafted the original manuscript. **M.V.O.L**, **V.M.S, R.G.B**, **D.B, M.T.G.G** contributed to the methodological refinement of the concept analysis, as well as the critical review regarding the international relevance and ethical framework. All authors have read and agreed to the published version of the manuscript.

## Funding

The authors have nothing to report.

## Ethics Statement

Ethical approval was not required for this study, as it is a purely theoretical and conceptual research project that did not involve human participants, animals, or personal clinical data.

## Conflicts of Interest

The authors declare no conflicts of interest.

## Data Availability

Data sharing is not applicable to this article, as no new empirical data were generated or analysed during the current study.
